# Diagnostic–Therapeutic Care Pathway in Chronic Constipation: AIGO (Italian Association of Gastroenterologists and Gastrointestinal Endoscopists) Position Paper

**DOI:** 10.3390/jcm15072571

**Published:** 2026-03-27

**Authors:** Maria Cristina Neri, Edda Battaglia, Francesca Galeazzi, Lucia d’Alba, Christian Lambiase, Paolo Usai Satta, Massimo Bellini, Gabrio Bassotti, on behalf of the AIGO Neurogastroenterology Commitee

**Affiliations:** 1 Pio Albergo Trivulzio Institute, 20146 Milan, Italy; 2 SC Gastroenterology ASL TO4, 10034 Chivasso, Italy; edda.battaglia@aslto4.piemonte.it; 3 Gastroenterology Unit, Padova University-Hospital, 35128 Padova, Italy; 4 Department of Gastroenterology & Endoscopy, San Camillo-Forlanini Hospital, 00152 Rome, Italy; 5 Gastrointestinal Unit, Department of Translational Research and New Technologies in Medicine and Surgery, University of Pisa, 56124 Pisa, Italymassimo.bellini@unipi.it (M.B.); 6 Regional Center for Functional and Motility Digestive Disorders, Azienda Ospedaliero Universitaria Pisana, 56124 Pisa, Italy; 7 Gastroenterology Unit, Brotzu Hospital, 09121 Cagliari, Italy; 8 Gastroenterology, Hepatology & Digestive Endoscopy Section, Department of Medicine and Surgery, University of Perugia, 06156 Perugia, Italy; gabassot@tin.it

**Keywords:** chronic constipation, constipation diagnosis, treatment, diagnostic–therapeutic care pathway, gut–brain interaction, anorectal manometry, pelvic floor dysfunction

## Abstract

Chronic constipation (CC) is one of the most common disorders of gut–brain interaction, affecting more than 11% of adults in Western countries, with higher prevalence in women and in the elderly. Despite its significant impact on quality of life, most patients self-manage their symptoms, while only a minority seek medical attention from general practitioners (GPs) or specialists. Proper assessment not only often requires a multidimensional approach but also accurate diagnostic and therapeutic pathways that define the exact role of GPs and specialists. This paper describes a comprehensive Diagnostic–Therapeutic Care Pathway (DTCP) for CC, focusing on the full spectrum of diagnostic and therapeutic methodologies required for accurate patient assessment and management. The pathway involves a primary care physician intervention phase, responsible for first-line diagnostic and therapeutic management and evaluation using objective parameters, as well as reassessment at appropriate time points to identify patients requiring further specialist evaluation. Advanced diagnostic methodologies are described as being performed in specialized gastroenterology or neurogastroenterology settings. These include colonic transit studies with radiopaque markers, high-resolution anorectal manometry, balloon expulsion testing, magnetic resonance imaging or conventional defecography, ultrasonography, and neurophysiological assessments such as anal sphincter EMG and pudendal nerve latency testing.

## 1. Introduction

Chronic constipation (CC) is one of the main disorders of gut–brain interaction most frequently encountered in clinical practice and affects over 11.7% (12–17%) of the adult population in Western countries, with greater frequency in women (F:M 2–3:1) and in the elderly [1], reaching up to 30–40% [2]. The prevalence of CC further increases when the assessment is based on patient self-report rather than on Rome criteria [3].

This disorder is characterized by a variable combination of different signs and symptoms. The criteria for making a formal diagnosis are defined by the Rome criteria, recently updated to the Rome V criteria (Table 1) [4].

Most patients self-manage CC without consulting their General Practitioner (GP) or a specialist. It is estimated that only about 30% of patients access a GP clinic, and only 10% consult a gastroenterologist [1].

In recent years, several scientific societies have produced protocols for the diagnosis and treatment of this condition, but their dissemination and application in clinical practice remain sporadic and insufficient [5]. International literature consistently shows that the proper evaluation of patients with constipation requires specific expertise and more time than is usually available during a primary-level gastroenterology visit, owing to the need for a complex, multidimensional approach that also considers comorbidities, including psychological and urogenital disorders.

Since it is neither possible nor appropriate for all patients with CC to be evaluated in specialist settings, shared pathways are needed that initially involve GPs and subsequently specialists in referral hospitals. This is essential to ensure more appropriate use of available resources and to guarantee each patient an optimal diagnostic and therapeutic approach, namely a Diagnostic–Therapeutic Care Pathway (DTCP).

It should also be noted that, with regard to the role of GPs in implementing the DTCP, the mandatory and systematic use of diagnostic definitions that rely on formally validated questionnaires or scoring systems increases workload and is often not feasible in local settings. A systematic diagnostic approach using validated scores, diaries, and questionnaires is instead required in gastroenterology outpatient clinics, where the most complex patients with an unsatisfactory therapeutic response are usually managed. If this approach becomes a shared practice between GPs and specialists, the burden of CC on referral centers will be reduced.

In this paper, we present a position statement on the practical diagnostic and therapeutic approach to CC, developed by the Neurogastroenterology Committee of the Italian Association of Hospital Gastroenterologists and Endoscopists (Associazione Italiana Gastroenterologi ed Endoscopisti Ospedalieri, AIGO).

## 2. Objectives of the DTCP

### 2.1. General Objective

To define the care pathway for patients with CC, from the initial diagnostic phase through management and follow-up, specifying the steps that fall under the responsibility of GPs and those that require specialist involvement.

### 2.2. Specific Objective

To define the diagnostic criteria applicable in the outpatient setting, the first-line therapeutic strategies to be implemented by GPs, and the pathway for identifying and managing patients with an “unsatisfactory therapeutic response”. This approach aims to promote more appropriate use of National Health System (NHS) resources for the diagnosis and treatment of CC, reduce waiting lists in referral hospitals, and improve the quality of life of patients with CC.

## 3. Role of the GP

GPs should perform an initial evaluation of patients with CC, using a structured approach to identify secondary causes and to select an appropriate first-line treatment.

### 3.1. First Evaluation and Diagnosis by GP

The first evaluation should integrate a detailed clinical history, rule out secondary and iatrogenic causes of constipation, identify alarm features that require prompt investigation or referral, and guide the selection of an appropriate first-line treatment.

#### 3.1.1. Patient History

Patient history represents the key element of the initial diagnostic assessment by the GP and guides subsequent diagnostic and therapeutic decision-making. Specifically, it should address:

- Global medical history and comorbidities (Table 2).
- Current and recent medications, with specific attention to drugs potentially causing constipation (iatrogenic constipation; see Table 3).
- Alarm features (e.g., weight loss, rectal bleeding, anemia, change in bowel habit with recent onset in older age; see Table 4)

#### 3.1.2. Definition of the Dietary Habits

Assessment of dietary habits should include the daily intake of fluids and fiber, the types of food consumed, and overall eating patterns.

#### 3.1.3. Definition of the Bowel Movements

It is important to properly investigate patient’s bowel habits. In detail:

- Weekly frequency of bowel movements;
- Stool consistency;
- Difficulty in evacuation and/or painful defecation;
- Sensation of incomplete evacuation and/or anorectal blockage;
- Use of manual maneuvers to facilitate defecation;
- Bloating and abdominal distention;
- Type of laxatives already taken (if any);
- Adequate intake of laxatives (if any).

Since symptom diaries have been shown to be a more reliable method for collecting daily data, their use is recommended even at the GP level, although this is not yet widely implemented in routine general practice [6].

#### 3.1.4. General Physical Examination

Physical examination must always be accurate and complete, with particular attention to abdominal tenderness, the presence of abdominal masses, possible hepatosplenomegaly, and the presence and quality of bowel sounds. Both central and peripheral neurological signs should also be carefully assessed.

**Table 3:** Drugs associated with constipation (adapted from Luo J et al. [7]).

Pharmaceutical Class	Molecules
Antacids	Calcium and Aluminum-containing
Antidiarrheal agents	Loperamide
Antihistamines	Diphenhydramine Doxylamine
Antiepileptics	Carbamazepine Phenytoin
Antiepileptics/Pain modulators	Gabapentin Pregabalin
Antiparkinson drugs	Benzatropine Trihexyphenidyl
Antipsychotics	Clozapine Thioridazine
Antispasmodics	Hyoscyamine Mebeverine
Beta blockers	Atenolol Propranolol
Calcium-channel blockers	Verapamil Diltiazem
Diuretics	Thiazide Loop diuretics
Supplements	Iron Calcium
Monoamine oxidase inhibitors	Phenelzine Selegiline
Nonsteroidal anti-inflammatory drugs	Ibuprofen Aspirin
Opiates	Morphine Oxycodone
Oral contraceptives	
Overactive bladder drugs	Oxybutynin
Sympathomimetics	Isoprenaline Phenylephrine
Tricyclic antidepressants	Amitriptyline Imipramine
GLP-1	Semaglutide Tirzepatide

**Table 4:** Red flags for Colonoscopy in CC patients.

Unexplained weight loss
Rectal bleeding (hematochezia)
Family history of bowel cancer
Fever
Iron-deficiency anemia
Age > 50 years (if no colonoscopy has been performed in the previous five years)

#### 3.1.5. Digital Rectal Examination (DRE)

DRE is a key diagnostic tool for detecting anorectal pathology and assessing pelvic floor dyssynergia and should always be performed when evaluating a patient with constipation.

**Figure 1:**
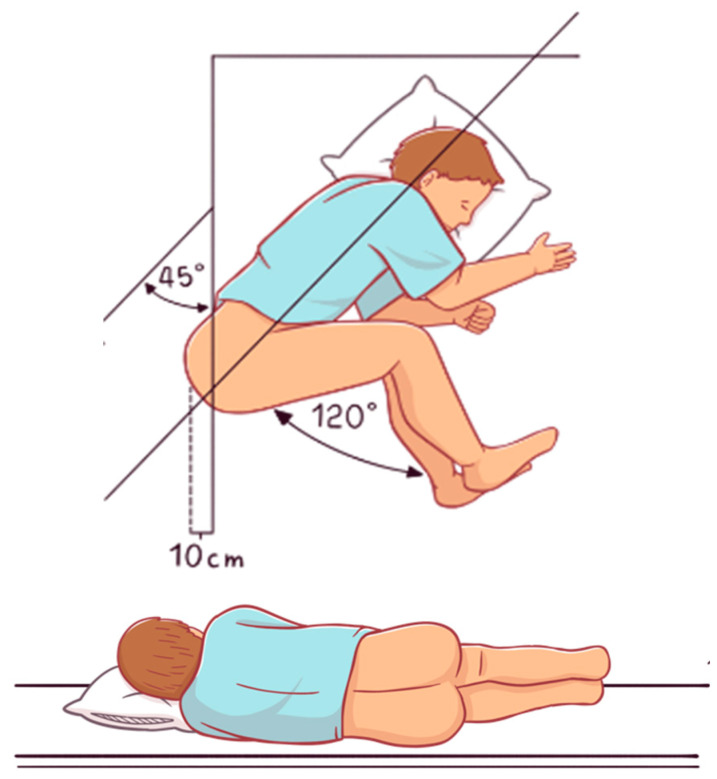
Position to perform DRE.

DRE can provide valuable information about the presence of stool in the rectal ampulla, stool consistency, and the presence of anorectal masses, hemorrhoids, anal fissures, rectal prolapse or rectoceles. The examination should be performed with the patient in the left lateral position, lying on the left side with the knees flexed toward the chest (Figure 1). Unfortunately, in routine GP practice, DRE is often difficult to perform consistently.

DRE should be performed with the patient in the left lateral decubitus position, with the knees flexed toward the chest (Figure 1). The examination should begin with inspection of the perineum. Simple observation may reveal anal malformations, erythematous or eroded perianal skin, fecal soiling, mucus or pus, scars, dermatitis, condylomas, fistulas, abscesses, external hemorrhoids, rectal and/or uterovaginal prolapse (full-thickness or mucosal), and a descended or descending perineum. Palpation of the perineum is then important to detect possible perineal masses (e.g., abscesses, fistulas) and to assess perineal sensitivity.

The physician may then proceed to the actual DRE, inserting the index finger into the rectum after adequate lubrication. Both a static and a dynamic assessment should be performed. During the static phase, the physician evaluates surface abnormalities of the anal canal and rectum, the presence of stool in the rectal ampulla, and the basal anal sphincter tone. Any pain elicited during the procedure should also be recorded. Pain on palpation of the Alcock canals may suggest pudendal neuropathy, whereas pain on palpation of the levator ani muscle may indicate a dyssynergic defecation [8].

During the dynamic phase of the DRE, the physician assesses possible anal sphincter weakness or evidence of dyssynergia. First, the patient is asked to squeeze as if trying to retain stool, and squeeze strength is evaluated. The patient is then asked to strain as if attempting to defecate. During this maneuver, the physician evaluates abdominal pushing effort with one hand placed on the abdomen and assesses anal relaxation with the examining finger. Failure to increase intra-abdominal pressure or inadequate relaxation of the anal canal has been associated with dyssynergic defecation [9]. The patient’s ability to expel the examiner’s index finger is also assessed.

At the end of the DRE, the color of any stool present in the rectal ampulla and the presence of blood, mucus or pus on the examining finger are evaluated. An Italian survey among gastroenterologists confirmed previous findings from the CHRO.CO.DI.T.E. study, showing that DRE is not frequently performed by Italian gastroenterologists, particularly among younger specialists [10,11].

#### 3.1.6. Laboratory Test

Laboratory (blood or fecal) tests should be performed in order to rule out secondary causes of CC, after carefully evaluating alarm signs and symptoms (i.e., full blood count, C-reactive protein, coeliac serology, thyroid stimulating hormone, serum electrolytes) [12].

#### 3.1.7. Colonoscopy

There is no clear evidence to support routine colonoscopy in patients with CC in primary care in the absence of alarm features [13]. However, based on the medical history and physical examination, the GP may request colonoscopy according to appropriateness criteria and in patients presenting with alarm symptoms (Table 4).

### 3.2. First Line-Therapy

After an accurate history, physical examination, and, when indicated, blood and instrumental tests to exclude secondary causes of constipation, the GP should prescribe first-line therapy. This includes lifestyle modification and dietary advice, with pharmacological treatment added if needed (Table 5).

#### Add-On Rescue Therapy

If the patient has previously used laxatives at an adequate dose and for a sufficient duration, but with unsatisfactory results, natural or synthetic agents, alone or in combination, may be prescribed as short-term add-on rescue therapy, limited to brief courses and discontinued once an adequate bowel habit has been restored. These include:

- Herbal stimulant laxative: Senna tablets (e.g., sennosides 7.5–8.6 mg/tab): 15–30 mg sennosides once daily at bedtime (≈2–4 standard 7.5–8.6 mg tablets). Use only for a few days, not >1 week without medical advice.
- Synthetic stimulant laxatives
  - Bisacodyl: 5–15 mg/day orally in a single dose, usually in the evening or after breakfast. Avoid use >1 week.
  - Sodium picosulfate: 5–7.5 mg once daily, preferably at bedtime to obtain effect the following morning. Start at 5 mg and titrate up.

### 3.3. Second Evaluation by GP

At the follow-up visit, at least two months after starting treatment, the GP assesses the response to therapy by re-evaluating the patient’s symptoms. The GP diagnoses an “unsatisfactory therapeutic response” if:

- The Bristol Stool Form Scale score is 1 or 2;
- Frequency of bowel movements is fewer than three per week.

**Figure 2:**
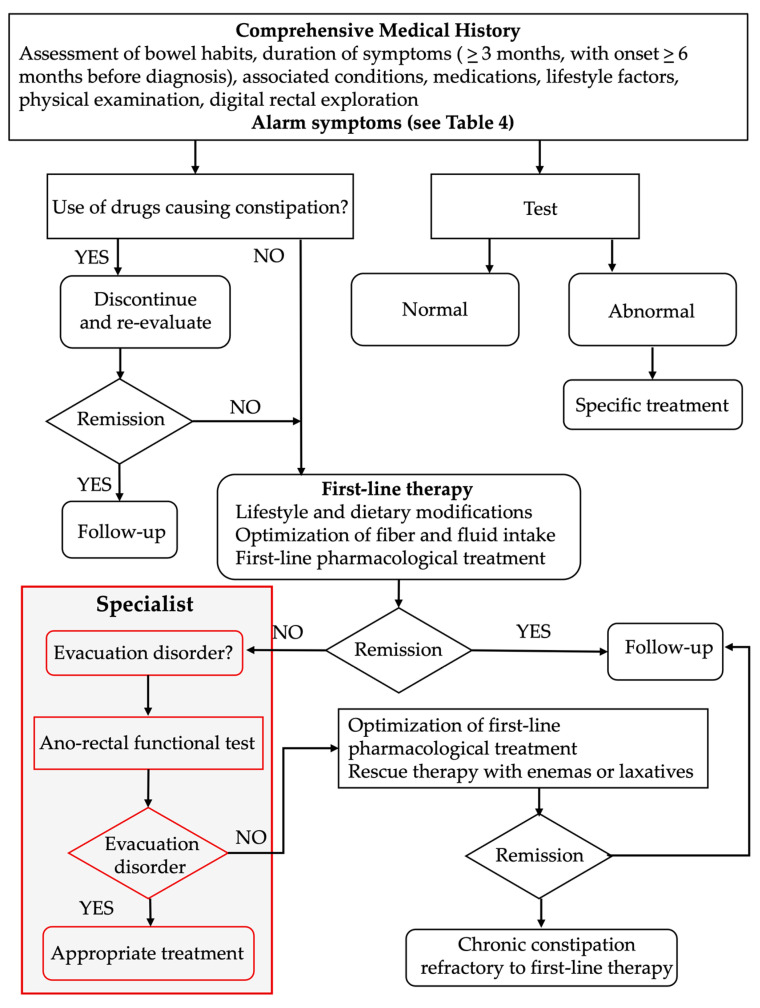
Diagnostic–therapeutic algorithm for CC: first approach by GP.

An “unsatisfactory” result for either of these parameters, after two adequate trials with laxatives, is sufficient to classify the patient as having constipation with an “unsatisfactory response to treatment” (see Figure 2).

## 4. From GP to Gastroenterologist

When the GP diagnoses an unsatisfactory therapeutic response, the patient should be referred to a specialized (neuro)gastroenterology clinic.

**Figure 3:**
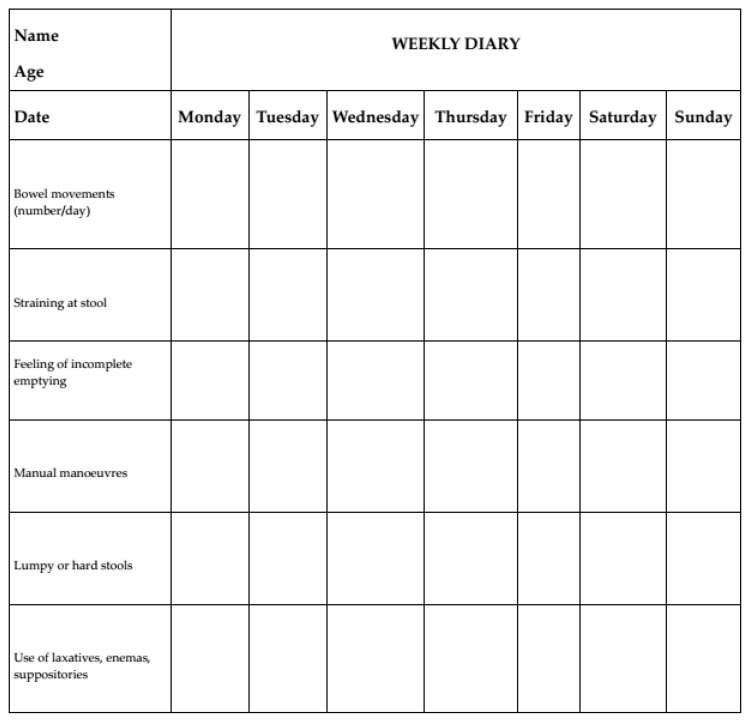
An example of defecation diary format.

The neurogastroenterologist or gastroenterologist will:

- Re-evaluate the clinical history and current clinical features;
- Rule out diseases that may be causing or contributing to constipation (see Table 2 and Table 3);
- Check adherence to treatment and reinforce lifestyle advice (see Table 5);
- Prescribe appropriate second-level investigations and adjust therapy;
- Ask the patient to complete a defecation diary using a validated or structured format, where available (see an example in Figure 3).

In case of a poor treatment outcome, the specialist should reassess the patient, after approximately 8 weeks, in order to prescribe appropriate second line therapy and/or imaging and anorectal function test (when appropriate and available).

### 4.1. Diagnostic Test Prescribed by Gastroenterologist

#### 4.1.1. Colonoscopy

Colonoscopy is suggested if it has never been performed before, according to appropriateness criteria and/or in the presence of alarm signs or symptoms (Table 4). In CC, colonoscopy has a low diagnostic yield. In patients with low-risk symptoms and no alarm features, the positive predictive value for colorectal cancer is ≤1.5% and for large polyps is approximately 3% [13]. Constipation alone, in the absence of alarm features, is not associated with an increased risk of colorectal cancer, and the diagnostic yield for significant pathology is low [14]. Therefore, colonoscopy should be reserved for CC patients who present with alarm features or for those over 50 years of age who have not undergone colorectal cancer screening [15,16].

#### 4.1.2. Computer Tomography (CT) Colonoscopy

CT colonoscopy is not routinely recommended in CC, unless alarm symptoms are present, and it is used as an alternative to colonoscopy and/or to complete the examination of the colon when colonoscopy is incomplete [15,16]. However, CT colonography may be useful for assessing colonic morphology and diameter when megacolon is suspected and a surgical approach is being considered.

#### 4.1.3. Colonic Transit Time with Radiopaque Markers

Colonic transit time with radiopaque markers can be used to distinguish normal-transit from slow-transit constipation. Diffuse retention of markers throughout the colon supports the diagnosis of slow-transit constipation, whereas accumulation of markers in the pelvic region supports the diagnostic hypothesis of an obstructed defecation syndrome [17,18]. Geographic and ethnic differences have been reported: studies from Europe describe a normal transit time of around 71 h, whereas studies from Asia and the Americas report values of approximately 49 and 44 h [19,20,21].

#### 4.1.4. Anorectal Manometry (ARM)

ARM is recommended to assess the functional integrity of the anal sphincter and to evaluate the possible presence of a dyssynergic defecation. It measures: (i) anal canal and rectal pressures at rest and during straining; (ii) the presence of the rectoanal inhibitory reflex (RAIR), which rules out Hirschsprung’s disease; and (iii) rectal sensitivity. The examination should be performed according to the standardized protocol proposed by the International Anorectal Physiology Working Group [22,23].

Compared with conventional ARM, high-resolution and high-definition ARM use a greater number of recording sites with multiple circumferential sensors, providing improved time–space resolution and making the technique more intuitive and reproducible than conventional manometry [24,25]. The London Classification incorporates findings from ARM, rectal sensation testing, and rectal evacuation tests into four diagnostic algorithms; however, these algorithms have not yet been fully validated in clinical practice [22]. Even so, they have been considered useful by the American Gastroenterological Association [26], the American College of Gastroenterology [27], and the American Society of Colon and Rectal Surgeons [28]. The results of ARM and related tests should always be interpreted in the context of the patient’s clinical characteristics and other investigational findings [29].

Of note, there is a wide overlap of findings between control and symptomatic subjects, making it difficult to interpret the results and to give a correct diagnosis [22,23,30,31,32].

#### 4.1.5. Balloon Expulsion Test (BET)

BET is a simple and inexpensive bedside screening procedure that identifies patients with pelvic floor dysfunction, such as anorectal dyssynergia, by assessing the subject’s ability to evacuate a rectal balloon [8,33,34].

The examination can be performed as part of ARM or as a stand-alone investigation and provides information on the time required to expel a 50 mL water-filled rectal balloon, which is usually 1–3 min in normal subjects; a longer expulsion time suggests impaired evacuation and may help predict response to biofeedback therapy [35,36,37]. BET has been reported to be highly sensitive and specific for detecting dyssynergic defecation, with impaired balloon expulsion observed in 23–67% of affected patients [38,39,40], although more recent data highlight important limitations in its stand-alone diagnostic performance. The test may be falsely normal in patients with pelvic laxity, such as those with a large rectocele or enterocele, or in patients who need to exert excessive straining to overcome increased outlet resistance. In addition, BET protocols and interpretation criteria are not yet standardized across centers [8,41].

#### 4.1.6. Ultrasound

In recent years, ultrasonography (intestinal ultrasound, IUS) has become a useful tool for assessing selected aspects of patients with CC. A recent position paper reported that colonic contents (solid or liquid) can be reliably visualized, the rectum can be identified after bladder filling, rectal diameter can be used as a marker of fecal impaction, and fecal loading can be documented [42]. Regarding transperineal ultrasound, the same document urged caution, as this technique requires a detailed knowledge of pelvic and rectal anatomy that may go beyond the standard competencies of IUS operators. The exact role of IUS in the evaluation of CC still needs to be defined [43,44].

#### 4.1.7. Conventional Defecography

Conventional defecography is useful to demonstrate structural (e.g., rectocele, prolapse) and functional (e.g., descending perineum syndrome, paradoxical contraction of the puborectalis muscle) abnormalities of the pelvic organs during evacuation (such as cystocele). It has a complementary role to physical examination, ARM and BET in cases where the diagnosis remains unclear or when structural abnormalities are suspected and surgical treatment is being considered [45].

#### 4.1.8. Dynamic Magnetic Resonance Defecography (MRD)

MRD has emerged over the past two decades as a key imaging modality for the assessment of pelvic floor disorders, with particular utility for the posterior compartment [46]. Unlike fluoroscopic defecography, MRD provides comprehensive anatomical and functional information without ionising radiation and offers superior soft-tissue contrast with multiplanar imaging [47]. This technique is especially valuable for diagnosing complex or combined conditions, including structural (e.g., rectocele, intussusception, enterocele) and functional (pelvic floor dyssynergia) abnormalities, thereby guiding optimal management [47,48]. However, MRD is not universally available and may be difficult to perform routinely in patients with CC.

#### 4.1.9. Standard or High-Resolution 3D Anal Ultrasound

When available, it can be used to assess the integrity of the anal sphincter complex, to detect septic complications (abscesses, fistulas, fissures), and to identify scars or sphincter injuries related to childbirth or surgery [10].

#### 4.1.10. Neurophysiological Tests

Neurophysiological tests should be reserved for selected cases, such as patients with known neuropathies or neurological diseases, and those being evaluated before surgical procedures or sacral neuromodulation [49,50].

#### 4.1.11. Anal Sphincter Electromyography (EMG)

When available, EMG can assess puborectalis relaxation during simulated defecation and is useful for detecting paradoxical contraction or failure of relaxation of the puborectalis muscle. Surface electrodes or a lubricated sponge electrode can be placed in the anal canal to measure muscle recruitment; needle electrodes placed in the anal canal are more painful and less well tolerated [28,51,52].

#### 4.1.12. Pudendal Nerve Terminal Motor Latency (PNTML)

PNTML testing, if available, evaluates the integrity of the pudendal nerves by measuring the distal motor latency. PNTML can be used to document chronic injury to the pudendal nerves, as can be seen in rectal prolapse and other constipation-associated conditions. PNTML is obtained using a nerve-stimulating and recording device mounted on a gloved finger (St Mark’s electrode), which measures the time from nerve stimulation to anal sphincter response. A normal PNTML is approximately 2.0 ms, and values >2.3 ms are considered abnormal; prolonged latency indicates pudendal neuropathy and may be observed in patients with fecal incontinence, constipation or rectal prolapse. It remains uncertain whether pudendal neuropathy is a cause of disordered defecation or a consequence of chronic straining [53,54].

### 4.2. Interpretation of the Diagnostic Work-Up

Rome V and current societal clinical practice guidelines show that a positive diagnosis of CC can be made in most patients based on typical clinical symptoms and the absence of alarm features without the need for extensive diagnostic testing: only digital rectal examination is recommended by all national guidelines and by the Rome V Committee. The Rome V Bowel Disorder Committee recommends performing anorectal function tests for patients who do not respond to therapeutic interventions; however, testing may be warranted earlier if there is strong suspicion of a defecatory disorder, such as abnormal findings on digital rectal examination [4]. Integration of clinical symptoms suggestive of difficult evacuation and anorectal function tests is necessary to achieve a correct diagnosis of dyssynergic defecation, according to the recently published Rome V Criteria (Table 6) [55].

A “probable” diagnosis of dyssynergic defecation according to Rome V criteria requires the presence of at least one of the symptoms of difficult defecation in at least 25% of defecations, together with an anorectal function test that shows evidence of dyssynergia. However, a “definitive” diagnosis requires two or more (≥2) concordant pathological tests [55].

Furthermore, Rome V introduced two new disorders of gut–brain interaction that may be present in patients with CC and may contribute to the genesis of symptoms: rectal hyposensitivity and rectal hypersensitivity. This update of the criteria emphasizes the importance of anorectal sensory dysfunction and the need for careful evaluation of this dysfunction during anorectal function testing [55].

**Figure 4:**
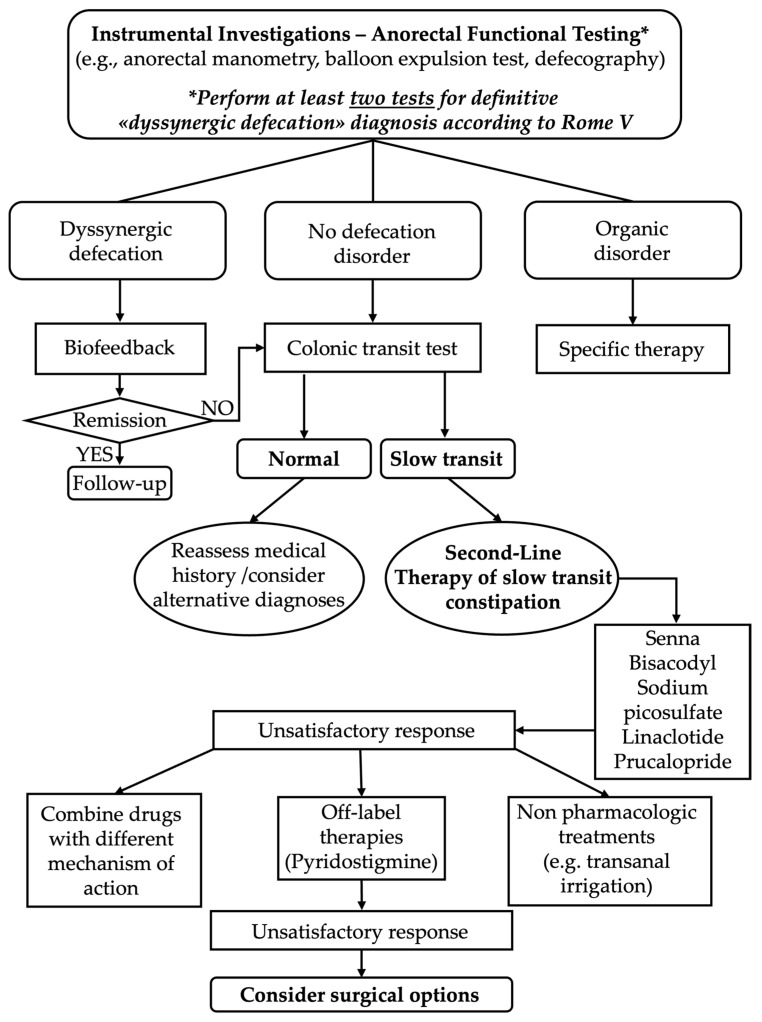
Diagnostic–Therapeutic Algorithm for Chronic Constipation Refractory to First-Line Therapy.

After the diagnostic tests and a specialist re-assessment, the patient can be classified into one of the three subtypes of CC mentioned above (with or without increased stool consistency):

1. Constipation with normal transit;
2. Constipation with slow transit;
3. Dyssynergic defecation, with or without slow transit and with or without significant structural abnormalities of the pelvic floor (Figure 4).

Based on the above diagnosis, the patient will then enter one of the following care pathways.

i. Reassessment and optimization of laxative and dietary therapy: for patients with chronic constipation without major pelvic floor or structural abnormalities.
ii. Rehabilitation therapy and psychological counselling: for patients with obstructed defecation, particularly those with documented pelvic floor dyssynergia or relevant psychosocial factors.
iii. Surgical evaluation: for patients with significant pelvic anatomical abnormalities or proven colonic inertia; these patients may also undergo rehabilitation therapy before or after surgery when ARM shows altered parameters.

### 4.3. Therapy Prescribed by Gastroenterologist

#### 4.3.1. Reassessment of First-Line Treatment

The first step is reassessing adherence to the prescribed therapy. Macrogol (starting from 10 g/day) should be taken daily, as a single or divided dose, for at least one month and then gradually reduced to the lowest effective dose, to be continued for at least 60 days. Enemas, micro-enemas or suppositories can be used 2–3 times per week when needed, or when no bowel movements have occurred for 3 days. A diagnosis of “unsatisfactory laxative response” should be made only after at least 1–2 months of adequate treatment.

#### 4.3.2. Add-On Laxatives

A combination therapy may be considered (macrogol plus enemas/micro-enemas). Irritant laxatives based on plant extracts (herbal products, senna tablets) should be used only as short-term rescue therapy for a few days. Synthetic stimulant laxatives, such as bisacodyl 5–15 mg/day and sodium picosulfate 5–7.5 mg/day, should likewise be taken for a few days and then reduced to alternate-day dosing or used as needed. A diagnosis of “unsatisfactory laxative response” should be made after at least one month of adequate treatment.

#### 4.3.3. Enterokinetic/Secretagogues

Prucalopride 2 mg/day (1 mg/day in older or frail patients), if this is what you intend) should be taken once daily for at least 15 days, after which the response should be reassessed; if satisfactory, treatment may be continued, with a one-week interruption every two weeks to reduce the risk of tachyphylaxis.

Linaclotide 290 µg once daily should be taken for at least one month; if the response is satisfactory on reassessment, treatment can be continued.

A diagnosis of “unsatisfactory response” to these agents should be made only after at least two months of adequate treatment.

#### 4.3.4. Enemas

The use of enemas is well established in the treatment of patients with CC and is generally employed as an adjunctive measure to enhance the effect of laxatives and to facilitate rectal emptying [56].

#### 4.3.5. Drugs for the Treatment of Opioid Constipation

Naldemedine, 200 µg once daily, is indicated in the treatment of opioid-induced constipation (OIC) in adult patients who are unresponsive to standard laxatives. It can also be used in combination with another type of laxative.

Naloxegol is a naloxone derivative that, however, penetrates the central nervous system to a limited extent compared to naloxone, blocking μ-opioid receptors in the intestine but not in the brain. By blocking the receptors in the intestinal tract, naloxegol, at a dose of 12.5 to 25 mg per day, reduces opioid-related constipating effects without interfering with the analgesic effects of opioids [57].

#### 4.3.6. Pelvic Floor Rehabilitation Therapy (If Available)

Pelvic floor rehabilitation (PFR) aims to restore abdomino-pelvic coordination, sphincter motor function, and rectal sensitivity in dyssynergic defecation. For this reason, it is reserved for selected patients with clear indications in whom conventional medical treatment (drugs, enemas, dietary measures) has failed [5].

The success of PFR depends on accurate diagnosis and careful patient selection based on targeted pathophysiological tests, and a multidisciplinary approach is strongly recommended, involving a gastroenterologist, colorectal surgeon/proctologist, gynecologist, urologist, neurologist, physiatrist, physiotherapist, and psychologist. A thorough cognitive and psychological assessment (with input from a psychologist and/or psychiatrist) is particularly important, as the presence of psychiatric disorders such as anxiety or depression may compromise the effectiveness of rehabilitation if these conditions are not addressed. Although the literature does not clearly define the optimal sequence of diagnostic tests, and clinical history and examination are often informative, the diagnosis should be confirmed by at least two investigations before starting PFR, depending on local availability: manometry plus balloon expulsion test, or manometry plus defecography, in order to define the underlying anatomical or functional abnormalities.

Before initiating PFR, incorrect lifestyle and dietary habits must be corrected. Toilet training should include a “ritualization” of defecation time, preferably in the morning after breakfast or after a meal, to exploit physiological reflexes such as the post-prandial colonic motor response and may be combined with low-volume (200–300 mL) warm-water cleansing enemas [5].

The PFR program is usually delivered in an outpatient rehabilitation setting in 6–10 sessions, using different techniques sequentially, and should be tailored to the individual patient according to clinical, radiological and manometric findings. At the end of the training program, the patient is referred back to the gastroenterologist for re-evaluation [58].

#### 4.3.7. Transanal Irrigation (TAI)

TAI may be considered in patients with refractory constipation who are dissatisfied with, or poorly adherent to, standard medical therapy. TAI uses dedicated devices that allow control of the timing, volume and pressure of water instilled into the anorectum and colon through a catheter or cone [59]. This technique can improve constipation-related symptoms and quality of life, including in patients with refractory CC associated with neurological disease or functional bowel disorders [60,61].

TAI can also be used as an adjunctive therapy, acting synergistically with laxatives; macrogol, in particular, may help soften stools and facilitate evacuation when combined with irrigation [59]. The clinical practice guidelines of the French Society of Coloproctology include TAI as an option for CC in cases where stimulant and/or prokinetic/secretagogue laxatives have failed [62]. However, the role of TAI in chronic constipation and dyssynergic defecation is not yet fully defined, and the evidence base remains limited, with heterogeneous response rates and relatively high discontinuation rates across studies.

Recent systematic reviews and expert opinions (e.g., Bassotti and colleagues, Tack et al., Bharucha and others) consider TAI a reasonable adjunct in carefully selected patients with refractory constipation, particularly before contemplating invasive surgical options, a position that is broadly aligned with the most recent AGA guidance on refractory constipation [63,64,65,66].

#### 4.3.8. Sacral Neuromodulation

Sacral neuromodulation cannot currently be recommended for the treatment of constipation, as available clinical studies have not demonstrated convincing evidence of efficacy [66].

#### 4.3.9. Surgical Therapy of Pelvic Anomalies

If investigations show structural abnormalities of the pelvic organs causing a significant obstruction to defecation, surgical/proctological evaluation is indicated. Surgical treatment should follow a multidisciplinary gastro-uro-proctological assessment, taking into account neuropsychological findings and the results of previous pelvic floor rehabilitation. From a surgical perspective, when a significant rectocele or rectal prolapse is identified, the following options may be considered:

- Delorme procedure for rectal prolapse/rectocele: mucosal resection of the prolapsed rectal segment (circular mucosectomy) with longitudinal plication of the muscular layer.
- STARR (Stapled Transanal Rectal Resection): transanal full-thickness resection of the rectal wall using circular staplers.

#### 4.3.10. Colon Resection Surgical Therapy

Colon resection surgical therapy for constipation is the last resort in the treatment of patients with CC, the medium- and long-term results remain controversial, partly because of the non-standardized preoperative assessment. It may be indicated in selected patients who do not respond to any kind of pharmacological treatment and whose quality of life is severely compromised. A multidisciplinary evaluation with a psychiatrist/psychologist is always recommended.

It is mandatory to refer for surgical evaluation patients who have previously undergone pathophysiological tests, including colon manometry with bisacodyl stimulation test [67], a test that can diagnose slow transit constipation due to *inertia coli* [68]. Such patients should undergo gastric and intestinal functional evaluation with gastric emptying test and gastrojejunal manometry to assess small bowel motor function, in order to exclude neuropathic or myopathic forms associated with constipation [69].

Such patients may be offered primarily TAI, Anterograde Continence Enema (ACE) or Malone’s Procedure, which is based on anterograde colonic irrigation through a cecostomy or appendicostomy (of the appendix/cecum or terminal ileum) or subtotal colectomy with rectal anastomosis, usually preceded by loop ileostomy (reversible if upper GI symptoms or abdominal pain or bloating persist).

## 5. Conclusions

In a system with limited resources and restricted availability of specialists, and in the context of rising healthcare demand, closer integration between GPs and specialists is essential to maintain an effective and efficient public healthcare system. In high-incidence and high-prevalence conditions that generate substantial costs for diagnostic tests and therapies, optimizing the use of available resources is the only way to ensure a consistent and coherent approach across different levels of care.

In the case of CC, establishing shared pathways with GPs that enable them to manage the initial phases of diagnosis and treatment independently can reduce unnecessary access to second- and third-level specialist clinics.

**Figure 5:**
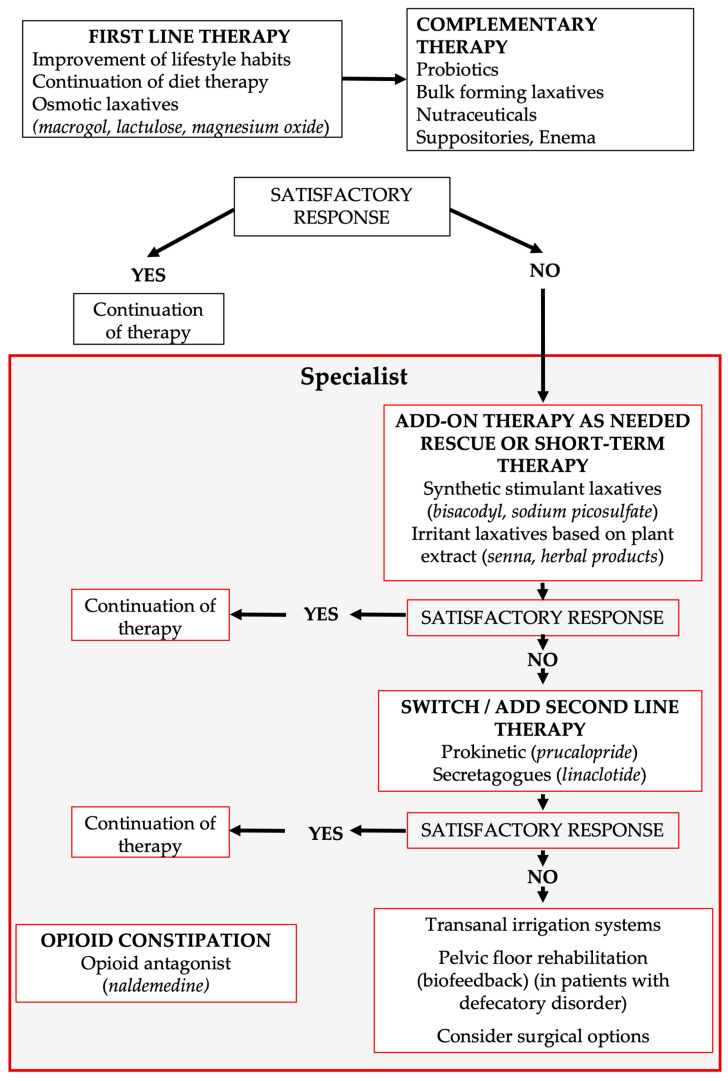
Therapeutic algorithm in chronic constipation.

The proposed DTCP for CC (Figure 5), developed in line with current international guidelines and the most recent Rome V criteria for disorders of gut–brain interaction, provides a structured framework that aligns the roles of GPs and specialists, promoting earlier, more appropriate management while reserving advanced diagnostics and complex therapies for truly refractory cases.

Widespread implementation and shared adoption of this pathway could improve patient outcomes and quality of life, while strengthening the integration and sustainability of the public healthcare system in the face of increasing demand.

## Figures and Tables

**Table 1:** Diagnostic Criteria for Chronic Constipation According to Rome V (adapted from Corsetti et al. [4]).

***1. Must include at least two of the following:***
a. Straining during more than 1/4 (25%) of bowel movements.
b. Hard or lumpy stools (1–2 on the Bristol Scale) during more than 1/4 (25%) of bowel movements.
c. Sensation of incomplete evacuation during more than 1/4 (25%) of bowel movements.
d. Sensation of anorectal obstruction/blockage during more than 1/4 (25%) of bowel movements.
e. Manual maneuvers to facilitate defecation during more than 1/4 (25%) of bowel movements.
f. Fewer than 3 spontaneous bowel movements per week
***2. Loose stools must be rarely present without the use of laxatives.***
***3. Criteria for irritable bowel syndrome must not be met***
The criteria must be met for at least 3 months, with symptoms having begun at least 6 months before diagnosis.

**Table 2:** Main secondary causes of constipation.

**Endocrine and metabolic disorders** Diabetes mellitus Hypercalcemia Hyperparathyroidism, Hypothyroidism Uremia	**Neurologic diseases** Autonomic neuropathy Cerebrovascular disease Hirschsprung’s disease Multiple sclerosis Parkinson’s disease Spinal cord injury Tumors
**Myopathic conditions** Amyloidosis Myotonic dystrophy Scleroderma	**Psychological conditions** Anxiety Depression Somatization
**Structural abnormalities** Anal fissures, strictures Colonic strictures Inflammatory bowel disease Rectal prolapse or rectocele	**Pregnancy**

**Table 5:** First line therapy for Chronic Constipation.

**GENERAL ADVICE**	
Lifestyle modification	Toilet training: set aside regular time, preferably in the morning after breakfast (to take advantage of the intestinal response to eating), using a squatting position (for example with a footstool) Drink at least 1.5–2 L of water per day
Dietary modification	Gradual intake of at least 30 g of fibers per day, starting with 5 g and increasing every week by 5 g until the target dose is reached. It may be suggested to eat fruits, green leafy vegetables, whole grains, legumes, nuts and seeds. Consider eating at least two green kiwifruits at breakfast, as tolerated. Increase fiber and fluids slowly to reduce side effects such as gas, bloating, abdominal distension and cramps. In case of side effects: a soluble fiber supplement such as psyllium (natural), methylcellulose (semisynthetic) or calcium polycarbophils (synthetic) is usually better tolerated. It is suggested to start with 5 g per day and to increase by 5 g each week until target dose is reached, if well tolerated.
**PHARMACOLOGICAL THERAPY**	
Macrogol (polyethylene glycol)	14–52 g/day as single or refracted doses for at least one month, then reduced to the lowest effective dose to be continued for at least 60 days
Lactulose	10–30 g as single or refracted doses for at least one month; then it should be reduced to the lowest effective dose and continued for at least 60 days
Enemas/microenemas suppository	2–3 times a week when needed or when there are no bowel movements after 3 days

**Table 6:** Diagnostic Criteria * for Dyssynergic Defecation (Adapted from Rao et al. [55]).

Must include all of the following:
1. The patient reports one or more symptoms suggestive of difficult evacuation (i.e., excessive straining, use of digital maneuvers to evacuate, sensation of anorectal blockage, and/or feeling of incomplete evacuation) with at least 25% of bowel movements and may satisfy diagnostic criteria for chronic constipation or irritable bowel syndrome.
2. During attempted defecation, they show features of impaired evacuation, as demonstrated by any one of the following 3 tests ** a. Reduced rectoanal pressure gradient or abnormal anorectal evacuation pattern with anorectal manometry b. Abnormal balloon expulsion test (BET) c. Impaired rectal evacuation with defecography

* Criteria fulfilled for the last 3 months with symptom onset at least 6 months prior to diagnosis. ** These abnormalities are identified by comparing the data obtained with age- and gender-appropriate normal values for that particular manometry system. Structural abnormalities such as rectocele, rectal mucosal intussusception and/or rectal sensory disorders may coexist with dyssynergic defecation.

## Data Availability

No new data were created or analyzed in this study.
